# Photobiological production of high-value pigments via compartmentalized co-cultures using Ca-alginate hydrogels

**DOI:** 10.1038/s41598-022-26437-y

**Published:** 2022-12-22

**Authors:** Runyu Zhao, Annesha Sengupta, Albern X. Tan, Ryan Whelan, Taylor Pinkerton, Javier Menasalvas, Thomas Eng, Aindrila Mukhopadhyay, Young-Shin Jun, Himadri B. Pakrasi, Yinjie J. Tang

**Affiliations:** 1 Department of Energy, Environmental & Chemical Engineering, Washington University, St. Louis, MO 63130 USA; 2 Department of Biology, Washington University, St. Louis, MO 63130 USA; 3 Biological Systems and Engineering Division, Lawrence Berkeley National Laboratory, 1 Cyclotron Rd, Berkeley, CA 94702 USA

**Keywords:** Symbiosis, Natural products, Gels and hydrogels

## Abstract

Engineered cyanobacterium *Synechococcus elongatus* can use light and CO_2_ to produce sucrose, making it a promising candidate for use in co-cultures with heterotrophic workhorses. However, this process is challenged by the mutual stresses generated from the multispecies microbial culture. Here we demonstrate an ecosystem where *S. elongatus* is freely grown in a photo-bioreactor (PBR) containing an engineered heterotrophic workhorse (either β-carotene-producing *Yarrowia lipolytica* or indigoidine-producing *Pseudomonas putida*) encapsulated in calcium-alginate hydrogel beads. The encapsulation prevents growth interference, allowing the cyanobacterial culture to produce high sucrose concentrations enabling the production of indigoidine and β-carotene in the heterotroph. Our experimental PBRs yielded an indigoidine titer of 7.5 g/L hydrogel and a β-carotene titer of 1.3 g/L hydrogel, amounts 15–22-fold higher than in a comparable co-culture without encapsulation. Moreover, ^13^C-metabolite analysis and protein overexpression tests indicated that the hydrogel beads provided a favorable microenvironment where the cell metabolism inside the hydrogel was comparable to that in a free culture. Finally, the heterotroph-containing hydrogels were easily harvested and dissolved by EDTA for product recovery, while the cyanobacterial culture itself could be reused for the next batch of immobilized heterotrophs. This co-cultivation and hydrogel encapsulation system is a successful demonstration of bioprocess optimization under photobioreactor conditions.

## Introduction

Photoautotrophs, such as the unicellular cyanobacterium *Synechococcus elongatus* UTEX 2973, enable conversion of CO_2_ to renewable products. For example, their high-flux sugar phosphate pathways can generate sucrose from CO_2_ as high as 2 g/L per day ^1^. However, genetic tools for cyanobacteria have lagged far behind those for classic heterotrophic hosts*.* Moreover, cyanobacteria have limited abilities to direct fluxes towards acetyl-CoA and the TCA cycle, which constrains their production of many molecules, such as biofuels and pharmaceutical products ^2–5^. To avoid this limitation, researchers have proposed microbial co-culture formats of autotrophic and heterotrophic species. These co-cultures can leverage the sucrose-secreting capability of *S. elongatus* UTEX 2973 to provide substrates for heterotrophic synthesis of high value products ^6,7^. Such consortia have three advantages: (1) converting CO_2_ to more diverse and valuable products, (2) easing subpopulation controls and growth optimization, and (3) enabling the division of labor between two hosts (one for CO_2_ fixation and the other for heterologous pathway engineering) ^8^. On the other hand, cyanobacterial photosynthesis may produce reactive oxygen species (ROS) that inhibit heterotroph viability, and heterotrophic growth may produce shading effects that reduce algal light harvesting ^9^. For example, in an artificial consortium of sucrose-secreting *S. elongatus* UTEX 2973 and 3-hydroxypropionic acid producing *Escherichia coli*, relatively low light intensity was used to reduce oxidative stresses, but this compromised the sucrose production rate ^7^. In general, stable and highly productive consortia are difficult to optimize unless the two species form a mutualistic growth relationship ^10^ with efficient ROS scavenging capability ^11^.

For this reason, cell immobilization has been attempted to develop stable co-culture systems for bioconversion ^12^. For cell encapsulation, hydrogel vessels have gained increasing attention owing to their ability to support cell growth ^13–15^. Particularly, alginate hydrogels are preferred materials because they are non-toxic, cheap, and environmentally benign ^13,16^. Moreover, the high porosity of alginate hydrogels allows nutrients and O_2_ to access immobilized cells by simple diffusion ^13,17,18^. Alginate encapsulation of *S. elongatus* 7942 was found to enhance its sucrose production and co-culture stability with polyhydroxybutyrate producing *Halomona boliviensis* over 5 months, and the encapsulated cyanobacteria resisted invasive microbial species during that time ^19^. Moreover, the same alginate-encapsulated sucrose-secreting cyanobacteria (within a barium–alginate hydrogel) and *Pseudomonas putida* formed a co-culture that both enhanced the degradation of an environmental pollutant, 2,4-dinitrotoluene, and synthesized the biopolymer polyhydroxyalkanoate ^6^. As this example demonstrates, hydrogel encapsulation connects individual species that possess different metabolic features, and it supports biological functions over long time periods. In previous studies, the products of engineered heterotrophs were secreted extracellularly, making them easy to separate. In cases where the products remain intracellular, product separation from consortia becomes difficult. Here, hydrogel encapsulation can advantageously trap specific producers and their products for further bio-separations.

Our recent paper ^20^ characterized Ca-alginate hydrogel for nutrient delivery to support cyanobacterial growth. In this study, Ca-alginate hydrogel was used to encapsulate the heterotrophic workhorses. In general, alginate hydrogels can be formed through crosslinking with a variety of cations. Two of the most used are barium and calcium. Barium produces stronger hydrogel and has a stronger binding affinity to alginate than calcium, and it has been used in making hydrogels to encapsulate cyanobacteria ^10,21,22^. However, barium in its soluble form is highly toxic ^23^. While a majority of the barium would be linked within the hydrogel, barium can leak during the microbial cultivation regime, creating a toxicity hazard when handling and disposing of spent medium ^24^. Additionally, barium strongly cross-links alginate, making it difficult to extract the insoluble product inside the hydrogel. In contrast, Ca-alginate hydrogels have lower binding strength than barium ^22^, so they are easily dissolved by EDTA for intracellular product recovery. For these reasons, we chose to use calcium as a non-toxic and low cost cross-linker for the alginate hydrogels to maintain hydrogel strength and longevity.

In this study, *P. putida* was used as a model bacterial factory ^25,26^ and *Y. lipolytica* was used as a model yeast factory ^27^. Complementing these workhorse microbes, *S. elongatus* UTEX 2973 has the highest sucrose secretion capability (1.9 g/L/day) of all cyanobacterial hosts. In our setup the autotrophic strain in the co-culture provides the sugar feedstock for the heterotrophic workhorses ^7,10^. However, for fast and efficient photosynthesis, UTEX 2973 requires high light intensity (> 800 μmol photons/m^2^/s) and high dissolved CO_2_ (3%) conditions ^28,29^. To provide sufficient light and CO_2_ to sustain high sucrose production, a co-culture where cyanobacteria are in free suspension and heterotrophic producers are encapsulated in Ca-alginate hydrogels would be highly beneficial. This approach also makes it convenient to scale up and reuse the photobioreactor (PBR). Additionally, the engineered *P. putida* and *Y. lipolytica* strains produce a blue compound (indigoidine, an industrial dye) and an orange compound (β-carotene) intracellularly ^30,31^. By encapsulating the *P. putida* or *Y. lipolytica* inside the Ca-alginate hydrogel, both the biomass and intracellular products will not interfere with cyanobacterial photosynthesis. Also, the hydrogel may protect light sensitive metabolites (e.g., β-carotene). Compared to previously demonstrated hydrogel consortiums using barium ^19^, the intracellular products inside the Ca-alginate hydrogel can be dissolved by EDTA and facilitate product extraction.

## Results and discussion

### Hydrogel compartmentalized co-culture (without sucrose utilization)

**Figure 1:**
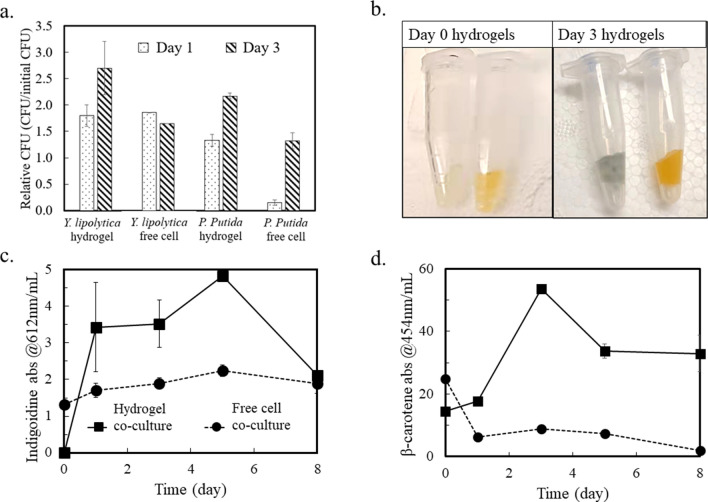
Co-culture of sucrose-producing *Synechococcus* and indigoidine-producing *P. putida* or β-carotene-producing *Y. lipolytica* (n = 2). (**a**) Relative CFUs of heterotrophic strains in co-culture. (**b**) Pictures of recovered hydrogels from co-culture system at day 0 and day 3. Left tubes, *P. putida*; right tubes, *Y. lipolytica*. (**c,d**) Absorbance of extracted indigoidine and β-carotene from co-culture. By using standard curves, the mass concentration of the two compounds can be estimated from the absorbance measurement (Supplementary Fig. S1). All data are averages of biological duplicates ± standard deviation. Some error bars are too small to be visible.

Cyanobacteria naturally supports heterotrophs because its “overflow metabolism” secretes amino acids (e.g., alanine and methionine ^11^), pyruvate and TCA metabolites under excess light energy input ^32–34^. Here, we separately co-cultured engineered *P. putida* and *Y. lipolytica* strains with *S. elongates* (Supplementary Table S1) with and without hydrogel compartmentation. Each co-culture system contained one heterotrophic species, either *P. putida* or *Y. lipolytica*. Since these heterotrophs cannot utilize sucrose, they survived by using cyanobacterial extracellular metabolites. Figure 1 shows heterotrophic growth and production from *P. putida/S. elongatus* co-cultures and *Y. lipolytica/S. elongatus* co-cultures, with and without hydrogel compartmentalization. Under the same initial cultivation condition, controlled by the multi-cultivator, hydrogel compartmentation shows protective effects on the growth of engineered *P. putida* and *Y. lipolytica*. Specifically, the heterotrophic strains inside the hydrogel produced much higher concentrations of the colored chemicals than those in free cell co-culture. Moreover, the living population of *P. putida* in free cell co-culture with *S. elongatus* drops dramatically from the beginning of co-culture to the end of day 1 (Fig. 1a). In contrast, the *P. putida* population in the hydrogel co-culture system increases continuously for three days. For *Y. lipolytica*, the living populations (based on colony forming units, CFU) in free cell co-culture and hydrogel co-culture with *S. elongatus* are similar in the first 2 days. This contrast indicates that *Y. lipolytica* are more tolerant to PBR stresses. Later, the *Y. lipolytica* population in free cell co-culture drops while that in the compartmentalized co-culture kept increasing. To enable two different heterotroph stains to grow and express engineered pathways in a co-culture, the chemical environment of the medium in system must be carefully chosen. As the base medium for cyanobacterial setup and co-culture, BG11 medium is not formulated for optimal growth of either heterotrophic partner (e.g., BG11 contains nitrate as a nitrogen source, whereas the medium for either heterotroph conventionally supplies ammonia ^35,36^). Additionally, the reactive oxygen species (ROS) generated as byproducts from photosynthesis is another stressor to *P. putida* and *Y. lipolytica*. By preventing the *P. putida* and *Y. lipolytica* encapsulated in hydrogels from direct contact with *S. elongatus*, the detrimental effects of cyanobacteria could be minimized so that the viability of the partner heterotroph is dramatically improved.

We further examined engineered heterotrophs’ productivity with cyanobacteria. Figure 1b shows *P. putida* and *Y. lipolytica* hydrogels on day 0 and day 3 of co-culturing. The color change indicates the accumulation of blue indigoidine and orange β-carotene. Measured at the respective absorbance wavelengths, the increases of product in the cell-free and hydrogel co-cultures are illustrated in Fig. 1c,d. The engineered heterotrophic microbes in hydrogels obviously generated a much higher concentration of indigoidine and β-carotene than the production in free cell co-cultures. Specifically, the cell density of *P. putida* in free cell co-culture increased by ~ 9 times from day 1 to day 3, from 2 × 10^7^ to 1.73 × 10^8^ CFUs/mL. However, the indigoidine production did not increase, and free *P. putida* cells lost their ability to generate the pigment under light and ROS exposure. In contrast, *P. putida* inside hydrogels maintained their productivity. On the other hand, *Y. lipolytica* could grow in free cell co-culture. β-carotene extracted from free cell co-cultures followed a descending trend in the 8-day cultivation. This observation was not surprising because the pigments were sensitive to ROS and light conditions (e.g., 50% of β-carotene naturally degrades within 48 h under fluorescent light) ^37^. In contrast, hydrogel could protect β-carotene from bleaching and increased its half-life (Fig. 1d).

### Development of a continuous co-culture platform (with sucrose utilization)

**Figure 2:**
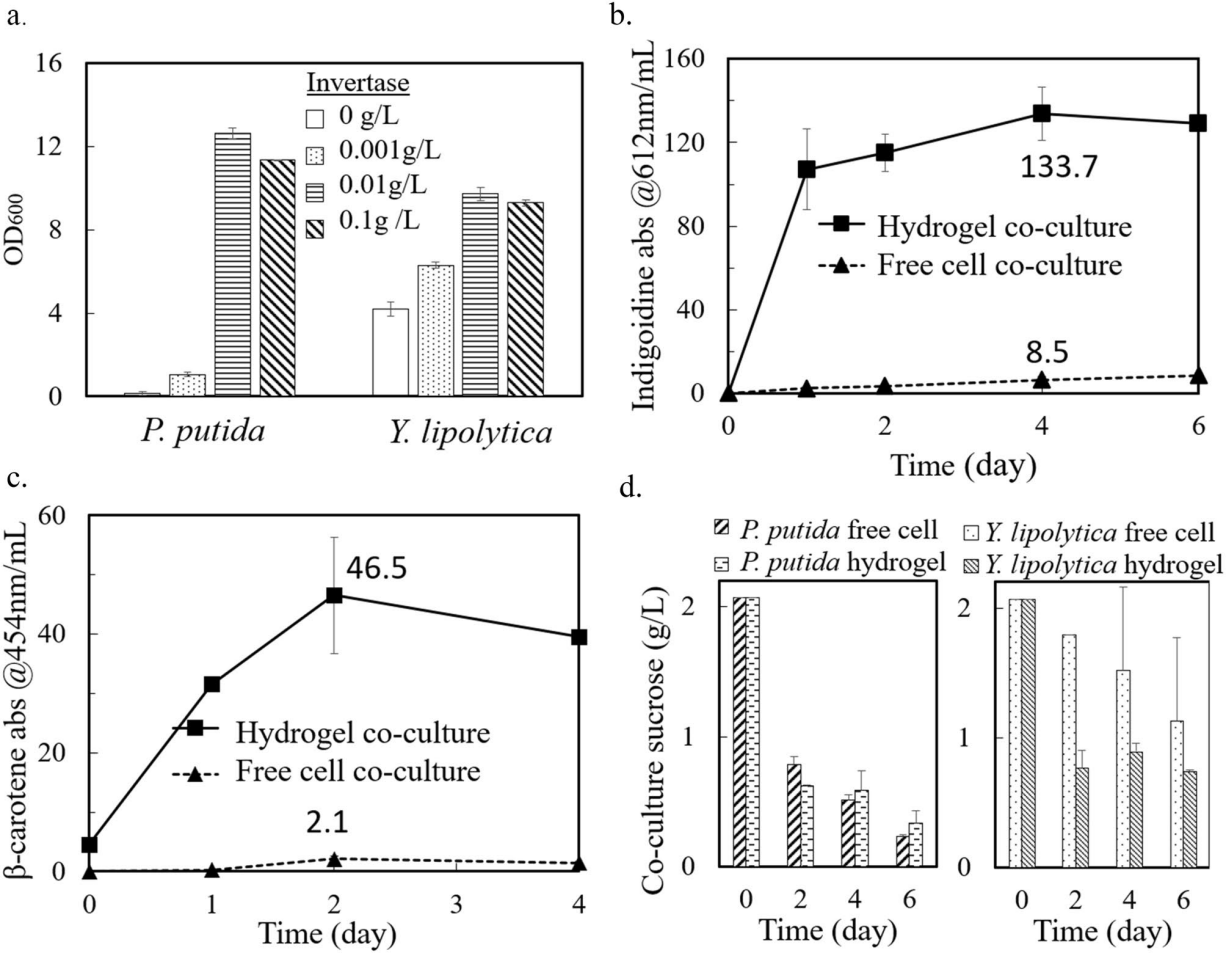
Axenic and co-culture heterotrophic growth/production with invertase. (**a**) OD_600_ of *P. putida* and *Y. lipolytica* growing in the defined media and 10 g/L sucrose at different concentrations of invertase (see “Methods” section). (**b**) Absorbance of extracted indigoidine at 612 nm from engineered *P. putida* over 6 days of co-culture. The peak production titer of indigoidine, on day 4, was 7.5 ± 0.7 g/L hydrogel. (**c**) Absorbance of extracted β-carotene from engineered *Y. lipolytica* over 4 days of co-culture. (**d**) Sucrose levels in *P. putida* and *Y. lipolytica* co-cultures. All data are averages of biological duplicates ± standard deviation. Some error bars are too small to be seen.

The first round of co-culture experiments was not set up for sucrose feeding, and both heterotrophs had to utilize overflow metabolites from cyanobacteria as nutrient sources. Because our *P. putida* and *Y. lipolytica* could not utilize sucrose, invertase, the enzyme hydrolyzing sucrose into fructose and glucose, was used to improve sucrose usage in axenic *P. putida* and *Y. lipolytica* cultures. Figure 2a displays the OD_600_ measurements of engineered *P. putida* and *Y. lipolytica* pure cultures after growing for 48 h in M9 and YNB media, respectively, with 10 g/L sucrose and different concentrations of invertase. Here, the addition of 0.001 g/L invertase was essential for bacterial growth, and 0.01 g/L invertase showed the highest growth promotion for both species.

After confirming the positive effect of invertase in boosting the growth of heterotrophic strains, biological duplicate co-culture tests with *S. elongatus* were set up with 0.01 g/L invertase. The measured absorbances of indigoidine and β-carotene are plotted in Fig. 2a,b. The peak *P. putida* indigoidine production in the hydrogel co-culture system was significantly boosted compared to the no-invertase co-culturing condition. During 6 days of cultivation, the indigoidine that accumulated in the hydrogel beads was protected from bleaching. The peak titer of indigoidine in hydrogel co-culture was 15 times that in free cell co-culture, and it reached over 7.5 ± 0.7 g/L hydrogel volume. Here, because hydrogel beads normally swell in an aqueous environment, the hydrogel volume refers to the initial volume. These results demonstrate the great advantage of our hydrogel co-culture for sensitive pigment production and harvesting. Similarly, the peak titer of β-carotene in hydrogel co-culture was 22 times greater than that in free cell co-culture, reaching 1.3 ± 0.3 g/L hydrogel volume using the sucrose generated by *S. elongatus*. Although the addition of invertase increased the β-carotene production titer by ~ 50%, the increase in β-carotene inside the hydrogel was not as significant as that of indigoidine (Figs. 1, 2). This observation was expected because the engineered *Y. lipolytica* strain requires a minimum threshold of exogenously supplied amino acids for β-carotene production ^36^. Because cyanobacterial culture contained only low levels of free extracellular amino acids, the residual rich nutrients in the medium could quickly become depleted would limit *Y. lipolytica* β-carotene production. In summary, the presence of invertase enabled the hydrogel co-culture to produce final products sequestered in hydrogels with comparable titers to glucose-based pure cultures ^25,31^.

The extracellular sucrose concentration from co-cultures was measured using an enzymatic kit and is shown in Fig. 2d. Pre-cultured *S. elongatus* reached 2.1 g/L sucrose before the heterotrophic species were added to the co-culture. For the *S. elongatus/P. putida* co-culture, the sucrose concentrations from the hydrogel co-culture and free cell co-culture did not show large differences throughout the 6 days of monitoring. In both co-cultures, the sucrose concentrations (including enzymatically hydrolyzed sucrose) dropped more than 50% within 48 h and then gradually decreased to below 0.4 g/L over 6 days. CFU results of the free cell co-culture showed significant *P. putida* growth with the help of invertase (data not shown), but the indigoidine accumulation in free *P. putida* was low, possibly due to ROS stresses and pigment bleaching when *P. putida* was fully mixed with *S. elongatus*. On the other hand, the yeast inside the hydrogel consumed moderately more sugar than in the free *S. elongatus/Y. lipolytica* co-culture. This result further demonstrated the usage of sugars from cyanobacteria by heterotrophic workhorses.

A previous study of compartmentalized co-cultures using hydrogel and sucrose-producing *S. elongatus* PCC 7942 reported their sucrose production in axenic culture as around 0.2 g/L in 48 h ^19^, where the cyanobacteria were encapsulated in barium-alginate beads. This study used a free culture of cyanobacteria but kept the heterotrophic workhorses inside the hydrogel, which resulted in 10 times more sucrose production. With better light and mass transfer conditions, we could optimize or scale up PBRs for cyanobacterial sucrose production to sustain workhorse growth and produce high value products inside the hydrogel.

### Reconciling incompatible growth conditions between two species

One of the key challenges for co-cultures is incompatible growth conditions for cyanobacteria and heterotrophs. For example, the optimal growth temperature for *P. putida* and *Y. lipolytica* is 30 °C, while *S. elongatus* UTEX 2973 grows best at 41 °C. Moreover, to maintain strain stability, engineered cyanobacteria require antibiotics that may interfere with partner strain growth. Here, engineered *S. elongatus* UTEX 2973 was grown in PBRs (without invertase), while heterotrophs were placed inside hydrogel beads. We examined heterotroph physiologies under different temperatures (30 °C vs. 35 °C) and antibiotic conditions (with or without 10 µg/mL kanamycin).

**Figure 3:**
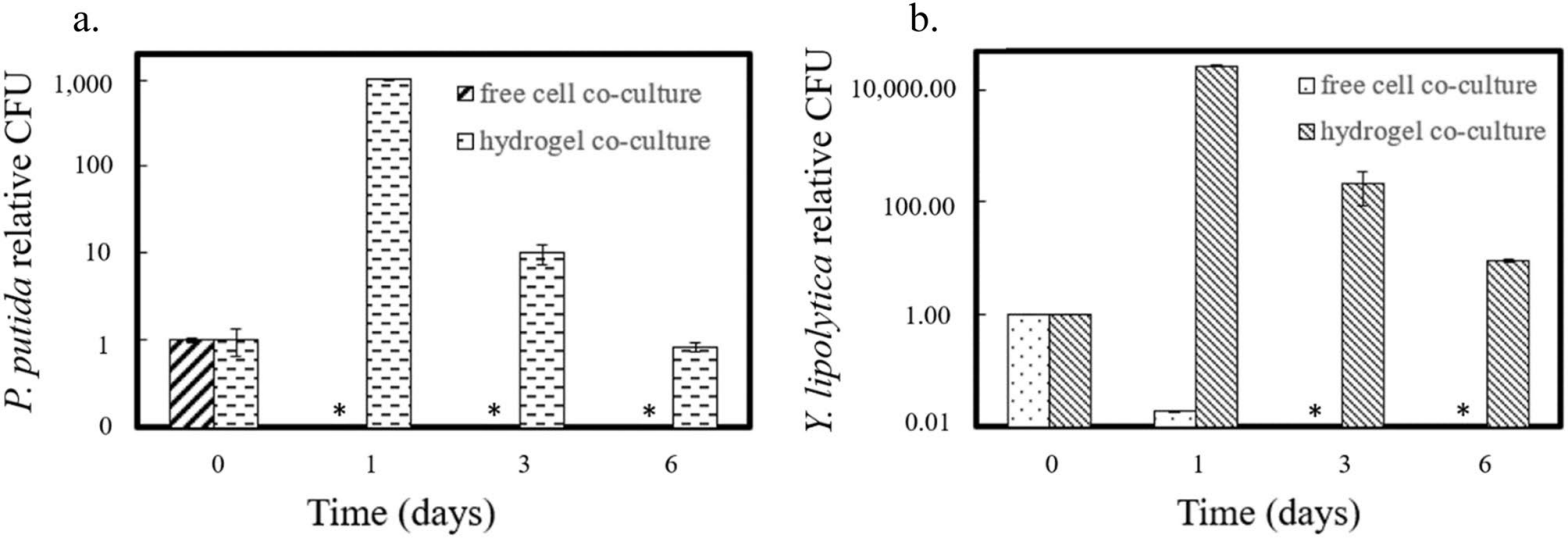
Relative heterotrophic cell population change in a stressful environment with high temperature and antibiotics. CFU values were normalized to the count at day 0. (**a**) Relative CFU counts of *P. putida* over 6 co-culture days. (**b**) Relative CFU of *P. putida* over six co-culture days. All data are averages of biological duplicates ± standard deviation. Some error bars are too small to see. Asterisks indicate no colony growth on plates.

In the stressful environment (35 °C and antibiotics), the hydrogels protected the bacteria and yeast cells capsulated inside (Fig. 3). Based on CFU measurements, free *P. putida* and *Y. lipolytica* cells without encapsulation were inviable under phototrophic co-culture conditions within a day. With hydrogel protection, both the *P. putida* and *Y. lipolytica* populations increased within the first day of co-culture. Both species survived inside the hydrogel for the 6 monitored days of co-culture. This extended survival demonstrates that hydrogels can protect heterotrophic cells in growth-challenging environments.

### Maximal growth of bacteria or yeast inside Ca-alginate hydrogels with rich media

**Figure 4:**
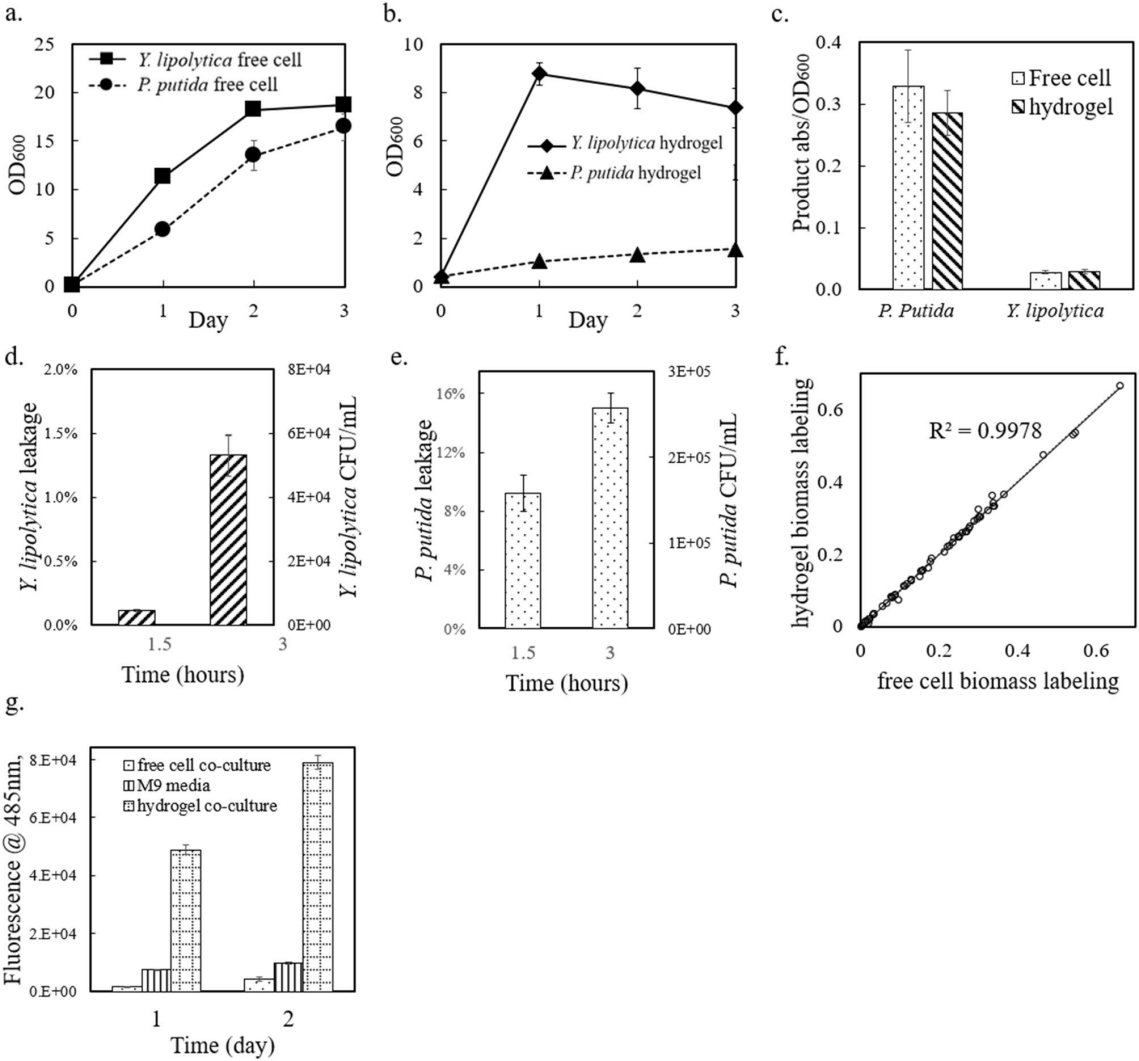
Heterotrophic culture behavior in rich media, with and without hydrogel compartmentation. (**a**) OD_600_ measurements of heterotrophic strains growing as free cells over three days. (**b**) OD_600_ measurements of heterotrophic strains growing in hydrogel compartmentation beads over 3 days. (**c**) Production of engineered *P. putida* (indigoidine) and *Y. lipolytica* (β-carotene) normalized to cell density. (**d**) *Y. lipolytica* cell leakage from hydrogel beads over 3 h, with shaking. (**e**) *P. putida* cell leakage from hydrogel beads over 3 h, with shaking. (**f**) 1,2-^13^C glucose labeling of *Y. lipolytica* proteinogenic amino acids from hydrogel and free cell cultures. The data points are from m/z = 57 peaks of GC–MS measurements of labeled amino acids. (**g**) Yellow fluorescent protein (YFP) fluorescence signal from engineered *E. coli* strain cultures (free cell co-culture, M9 media pure culture, and hydrogel co-culture). Data from plot (**a–e,g**) are averages of biological triplicates ± standard deviation. Some error bars are too small to be seen.

To examine cell growth inside hydrogels, hydrogel beads with encapsulated cells were washed and then added to rich aqueous media (LB or YPD) with inducers as necessary. Figure 4a,b show that the engineered *P. putida* and *Y. lipolytica* strains grew inside the hydrogel beads and in the free medium. In the stationary phase, the OD_600_ of *P. putida* inside the hydrogels was 1.5 (after EDTA dissolution of the hydrogel), and that of the free *P. putida* culture was ~ 14. As for *Y. lipolytica*, the OD_600_ inside the hydrogel beads reached 7, while the OD_600_ of free yeast culture was ~ 19. In general, the free monoculture grew faster than the immobilized cells. *P. putida* inside the hydrogel produced only ~ 10% of the biomass in the free LB culture. In contrast, *Y. lipolytica* inside the hydrogel reached > 40% of the biomass in the free YPD culture. Inside the hydrogel, the normalized product concentration by OD_600_ was similar to that in the free monocultures (Fig. 4c). These results confirmed that *P. putida* and *Y. lipolytica* cells maintained their productivity inside hydrogel microenvironments. Ca-alginate hydrogels are advantageous for the nutrient diffusion, but they may also have cell leakage. To investigate this problem, hydrogel beads with encapsulated *P. putida* or *Y. lipolytica* cells were washed and then incubated in nutrient-free 0.9% NaCl solution. The saline water was then sampled and spread on agar plates to count leaked cells. As illustrated in Fig. 4d,e, the numbers of leaked *P. putida* and *Y. lipolytica* cells increased with shaking for 1.5 h to 3 h, showing that the microbial cells were not completely immobilized inside the hydrogel microstructure. *P. putida,* being smaller than *Y. lipolytica*, more easily escaped from the hydrogel. Because heterotrophic species survived poorly in free co-culture without hydrogel protection, understanding and optimizing the microstructure of hydrogel beads is important for both restricting cell leakage and maintaining sufficient mass transfer.

### Metabolic analyses of spatially distinct microbes present in synthetic consortia

Hydrogel technology provides a convenient way to study individual microbial species’ physiologies in a consortium. First, we investigated the heterotroph’s metabolism inside the hydrogel via isotope tracing ^38^. Specifically, the metabolic flux distributions in the heterotroph cells could be inferred from ^13^C-labeling patterns of their proteinogenic amino acids. Using 1,2 position labeled glucose, we performed an isotropic labeling experiment on wild type *Y. lipolytica* that was grown either in free cell culture or inside the hydrogel. The biomass was harvested during the mid-exponential growth phase, and the labeling data in proteinogenic amino acids (i.e., ^13^C-fingerprints) were analyzed ^39^. Figure 4f shows the ^13^C labeled fraction from the [M-57] MS peak of each amino acid from both the hydrogel-immobilized cells and free cells (numerical results are included in Supplementary Material Table S2). The labeling results in the exponential growth phases are almost the same (R^2^ > 0.99), showing that during pseudo steady growth phase, the metabolic flux distributions of immobilized *Y. lipolytica* cells were almost identical to those of the free cell cultures. Therefore, the hydrogel encapsulation process and crosslinking structure did not compromise the metabolic activity of the yeast workhorse.

To further explore the possibility to generalize the Ca-alginate hydrogel co-culture method to other heterotrophic species as well as their heterologous protein expressions, an engineered *E. coli* strain expressing yellow fluorescent protein (YFP) was tested using the same co-culture step-up (with sucrose utilization). The advantage of hydrogel compartmentation held for this model bacterial species. Comparing the total fluorescent signals among free cell co-culture, hydrogel co-culture, and axenic bacterial culture using M9 media, the hydrogel co-culture shows the highest fluorescence while the axenic bacterial culture showed consistently increasing signal (Fig. 4g). The normalized fluorescence per cell from the hydrogel co-culture is even higher than that from axenic condition. As for growth, the CFU counting results from day 2 showed that the number of living cells from hydrogel co-culture is 10,000 times of that from free cell co-culture. The higher protein expression and healthier growth from hydrogel co-culture *E. coli* can be explained by the continuous supply of sugar from the autotrophic module and the metabolite overflow, such as amino acids, that promotes the *E. coli* growth and YFP expression inside hydrogel. Hence, the protein expression level from cells capsulated in hydrogel co-culture is not compromised by the hydrogel fabrication and co-culture stress.

## Conclusions

This study demonstrated the production of high-value pigments and chemicals using a hydrogel-enabled phototroph-heterotroph co-culture consortium. Immobilizing the heterotrophic strains inside Ca-alginate hydrogel beads allowed the workhorse strains *P. putida* and *Y. lipolytica* to take advantage of the high sucrose production from engineered cyanobacteria in the free medium and produce high product titers inside the beads. The calcium alginate matrix also created a protective micro-environment that protected the heterotrophic species and sensitive pigment products from ROS released by cyanobacteria or from environmental stresses, such as high temperature and antibiotics. This methodology can be applied to more complex consortia involving more than two species. The hydrogel-encapsulated products were easily harvested from the photobioreactor, and the free cyanobacterial culture can be continuously resupplied with new batches of heterotroph-containing hydrogel beads. Furthermore, the hydrogel technology may facilitate metabolic analysis (e.g., ^13^C-metabolomics or proteomics) from spatially distinct microbes present in synthetic consortia.

While our study is an ideal case study for process optimization to enable the use of photosynthetic organisms, there are still several challenges that remain. First, engineering cyanobacteria or heterotrophs for invertase secretion may be advantageous over the addition of commercially produced invertase to the growth medium. Expression of a sucrose invertase has been demonstrated in *P. putida* but its secretion is still challenging ^40^. This strategy will have to be carefully optimized due to the potential tradeoff among final product titers, metabolic burdens for enzyme expressions, and invertase secretion. Second, phototrophic coculture still had lower titer than heterotrophic fermentations using rich medium. Our BG11 medium for cyanobacterial growth was suboptimal for engineered strains that require rich medium to sustain heterologous biosynthesis. In future work, the culture media and cultivation parameters should be further optimized. For example, the cyanobacteria could be engineered to overflow various nutrients to supply workhorse for optimal bioproduction.

## Methods

### Materials and strains

Sodium alginate (FCC grade) was purchased from Spectrum Chemicals (New Brunswick, NJ, USA). Reagent grade invertase was purchased from Carolina Biological (Burlington, NC, USA). Yeast extract, peptone, and agar for media were purchased from BD Biosciences (Franklin Lakes, NJ, USA). 2000X trace element was purchased from Teknova (Hollister, CA, USA). Amino acid supplements were purchased from US Biological Life Sciences (Salem, MA, USA). All other chemicals were purchased from Sigma-Aldrich (St. Louis, MO, USA). The sucrose producing *S. elongatus* strain and a β-carotene producing *Y. lipolytica* strain (provided by Arch Innotek, St. Louis, USA) were described in previous studies ^1,36^, respectively. The blue pigment producing engineered *P. putida* strain was engineered at the Lawrence Berkeley National Laboratory and this engineered strain was unable to use arabinose ^25,41,42^. The yellow fluorescent protein expressing *E. coli* is constructed from the Pakrasi lab (Supplementary Table S1).

### Growth conditions

For sucrose production, engineered *S. elongatus* UTEX 2973 was first grown in BG11 medium for 48 h at 38 °C (250 μmol photons/m^2^/s^1^) in an AlgaeTron growth chamber (Photon Systems Instruments, Czech Republic), and then diluted tenfold in BG11 with 150 mM NaCl and 1 mM IPTG to grow for another 3 days at 38 °C (900 μmol photons/m^2^/s^1^) on PBRs (Multi-cultivator MC 1000-OD, Photon Systems Instruments, Czech Republic) before co-culture ^1^. In brief, BG11 media was modified from the UTEX Culture Collection, containing 17.6 mM NaNO_3_, 0.23 mM K_2_HPO_4_, 0.3 mM MgSO_4_, 0.24 mM CaCl_2_, 0.021 mM ferric ammonium citrate, 0.0027 mM Na_2_EDTA, 0.19 mM Na_2_CO_3_, and 10 mM N-[Tris(hydroxymethyl)methyl]-2-aminoethanesulfonic acid (TES) buffer. Around 1% v/v CO_2_ was maintained in the cyanobacterial media by bubbling CO_2_ into the sample tubes. The mixing was controlled by bubbling. *Y. lipolytica* seed cultures were grown in YPD medium (per liter: 10 g yeast extract, 20 g peptone, 20 g of glucose). A single colony (from YPD plates) was used to inoculate seed cultures in YPD and grown overnight at 30 °C in shaking flasks at 250 rpm. *P. putida* seed cultures (from single colonies picked from LB plates) were grown overnight in LB medium (per liter: 10 g tryptone, 5 g yeast extract, 10 NaCl) in shaking flasks at 30 °C at 250 rpm. *E. coli* seed cultures (from single colonies picked from LB plates) were grown overnight in LB medium with 20 µg/mL kanamycin in shaking flasks at 37 °C at 250 rpm. For YFP expression in axenic culture, engineered *E. coli* was grown in M9 media (per liter, 2 g (NH_4_)_2_SO_4_, 6.8 g Na_2_HPO4, 3 g KH_2_PO_4_, 0.5 g NaCl, 1 mL trace elements solution, 100 μL 1 M CaCl2, 2 mL 1 M MgSO4) supplemented with 2 g/L sucrose, 0.01 g/L invertase, and 20 µg/L kanamycin. For leakage test, 3 mL cell encapsulating hydrogels were added into 5 mL 0.9% NaCl solution. The mixtures were shaken in sample tubes at 30 °C at 250 rpm before CFU counting.

For the invertase tests of *Y. lipolytica*, amino acid was supplemented to BG11 media for β-carotene production. The amino acid supplements added each of 20 amino acids to the medium at 0.04 g/L (except for leucine, 0.2 g/L); uracil and inositol at 0.04 g/L; adenine at 0.01 g/L; and p-Aminobenzoic acid at 0.004 g/L.

### Hydrogel fabrication and co-culture

**Figure 5:**
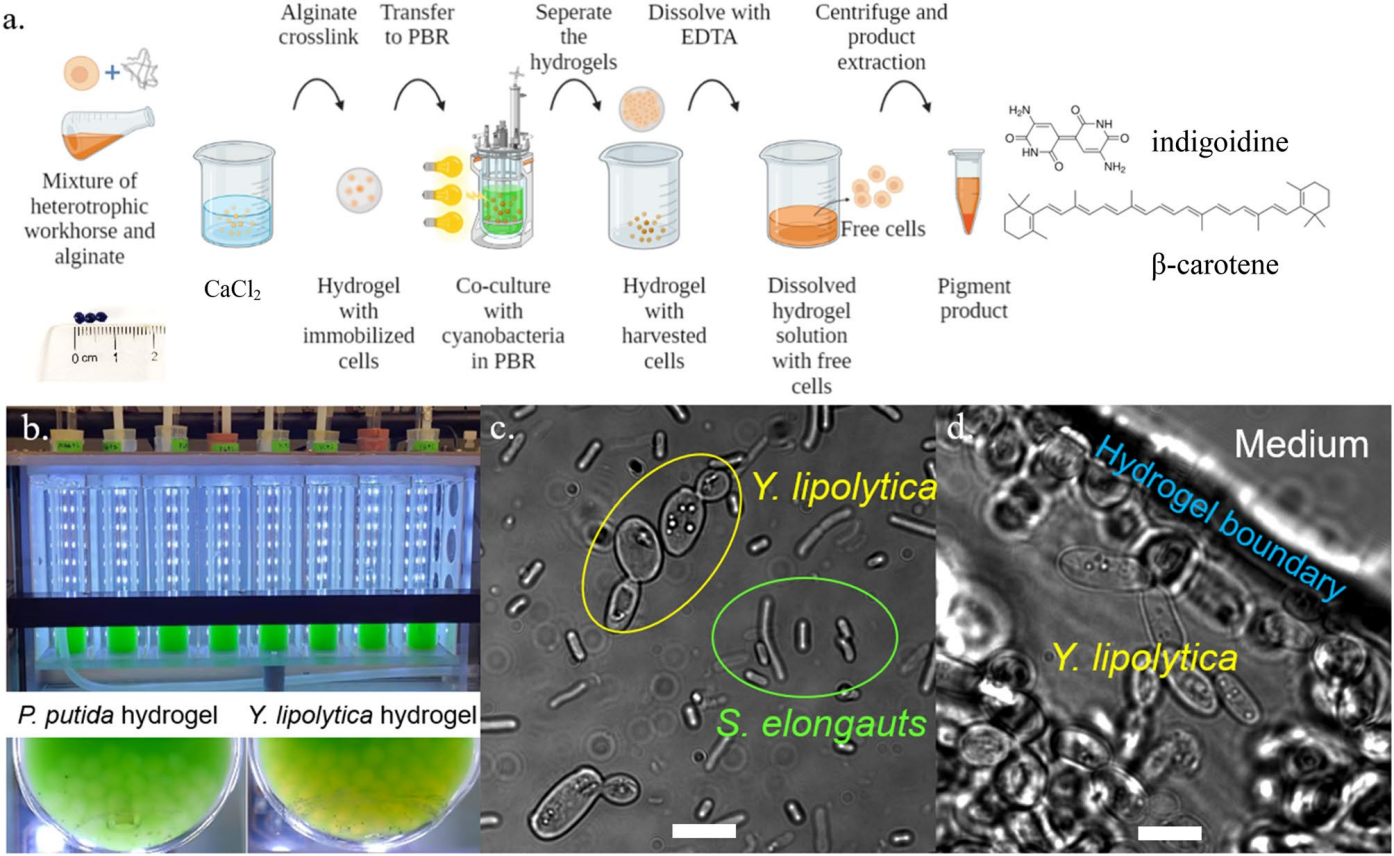
Co-culture illustration diagram, multi-cultivator setup, and microscope images of co-culture and hydrogel. (**a**) Schematic illustration of co-culture bioproduction process. Created with BioRender.com. (**b**) Picture of the multi cultivator with *P. putida* and *Y. lipolytica* embedded hydrogels. (**c**) Optical microscope image of free cell *S. elongatus/Y. lipolytica* co-culture. (**d**) Optical bright field microscope image of hydrogel free cell *S. elongatus*/*Y. lipolytica* co-culture, × 100 magnification. Scale bars are 10 µm.

A 0.6% (w/w) sodium alginate solution mixed with *P. putida* or *Y. lipolytica* cell culture (OD_600_ = 2) was dripped into 100 mM calcium chloride gelation baths. The sodium alginate drip was driven by a syringe pump at a rate of 5 mL/min from a height of 10 cm above the bath, with magnetic stirring at 100 rpm. The resulting hydrogel beads were soaked statically in the calcium chloride bath for 2 h and then washed with DI water three times to remove unreacted calcium chloride. The fabricated hydrogels were spherical with diameter around 3 mm. Each bead contains about 10 million *P. putida* cells or 1 million *Y. lipolytica* cells. The workhorse (*P. putida* or *Y. lipolytica)* embedded hydrogel beads were then soaked in their respective rich media to resume their growth. The immobilized heterotrophs were added to the *S. elongatus* cultivation cultures to make *P. putida/S. elongatus* and *Y. lipolytica/S. elongatus* co-cultures. 3 mL heterotrophic hydrogel beads or cell culture was added to 27 mL cyanobacteria culture in tubes of a multi-cultivator at 30 °C under 800 μmol photons/m^2^/s^1^. 5 g/L arabinose was added to the *P. putida* co-culture to induce the production of indigoidine. For stress study of co-culture, we set the multi-cultivation temperature to 35 °C and added 10 µg/L kanamycin to the co-cultures. The picture of the co-culture system is shown in Fig. 5b. Figure 5c shows the microscope image of free cell co-culture of *S. elongatus* and *Y. lipolytica* (× 100 magnification). The microscope image of resulted cell-capsulated hydrogel is shown in Fig. 5d. Fluorescent image of indigoidine producing *P. putida* images were taken using the 4ʹ,6-diamidino-2-phenylindole (DAPI) filter, due to the fluorescence of indigoidine with 415 nm excitation wavelength and 520 nm emission wavelength ^43^. The fluorescent filter cube has excitation filter at 375 nm with 28 nm bandwidth and barrier filter at 460 nm with 50 nm bandwidth. Images of free cell co-culture and hydrogel on day 1, and hydrogel on day 4 are shown in Supplementary Fig. S2. The *E. coli/S. elongatus* co-cultures were constructed in similar way as described earlier in this session, except that the cultivation temperature was set at 38 °C and 20 µg/L kanamycin was added to the co-culture.

### Growth and production quantification

Growth of the three microbe strains was monitored by measuring the OD_600_ (*P. putida* or *Y. lipolytica*) and OD_730_ (cyanobacteria) on a Cary 60 UV–Vis Spectrometer. Strain survival was quantified as colony forming units (LB plate for *P. putida*, YPD plate for *Y. lipolytica*, and BG11 plate for *S. elongatus*) from liquid culture or mechanically crushed hydrogels. Sucrose and glucose concentrations in co-culture were measured using colorimetric enzyme kits, and absorption was measured on a Cary 60 UV–Vis Spectrometer. To measure the β-carotene content, 100 mL *Y. lipolytica* cultures were pelleted at 24,000×*g* and the supernatant was discarded. The biomass was resuspended in 1 mL hexane and vortexed with glass beads until lysed. β-carotene titer was quantified via absorbance at 454 nm on a Tecan Infinite M200 Pro Microplate Reader ^36^. Because the measurement of yeast β-carotene from free cell co-culture could be complicated by native cyanobacterial pigments, we measured the background absorbance from pure *S. elongatus* culture. Then the β-carotene absorbance readings from the free cell co-culture were subtracted by the background readings. The entire hydrogel co-culture production process is illustrated in Fig. 5a.

Indigoidine production and quantification were performed as previously described with slight modifications ^25^. Briefly, 100 µL of liquid culture was pelleted at 24,000×*g* for 2 min. The supernatant was discarded, and 500 µL of dimethyl sulfoxide (DMSO) was used to resuspend and extract the indigoidine via vortexing (10 min). Additional DMSO was added if the pellets were not fully dissolved. Absorption intensities at 612 nm were measured with a Tecan Infinite M200 Pro Microplate Reader. Standard curves of β-carotene and indigoidine were obtained by solubilization of the pure color chemicals in their perspective solvents, serial dilution, and absorption measurement with the Tecan microplate reader (Supplementary File).

For extraction of products from hydrogel encapsulated samples, 25 µL of 500 mM EDTA was added to 10 recovered hydrogel beads (total volume ~ 1.5 mL) to dissolve the structure. Once solubilized, the resulting crude or β-carotene or indigoidine was estimated with absorbance after hexane or DMSO solubilization as for a standard cell pellet. Blank samples were prepared using hydrogels without microbes through the same extraction procedure. The blank values were subtracted from raw readings to avoid noise.

### Determination of colony forming units (CFUs)

Agar plates were prepared for microbial growth (BG11 plates for *S. elongatus,* YPD plates for *Y. lipolytica*, LB plates for *P. putida*). A serial dilution cultures were done in a 96 well plate using sterilized water as dilutant. For plating, 10 μL aliquot of each dilution was transferred onto the agar plates. Agar plate lids were kept open in laminar flow hood until all of the liquid had dried. The plates were incubated at 30 °C upside down until single colonies were clearly visible. The number of colonies were counted, and original titers were calculated based on total volume plated, and dilution. To determine the CFUs in the hydrogels, the hydrogels were mechanically broken into pieces by pipetting up and down using 1 mL pipette. The CFUs from broken hydrogels were estimated as for a free cell culture.

### ^13^C labeling of proteinogenic amino acids

Overnight cultures of wildtype *Y. lipolytica* were subcultured 0.3% v/v into respective minimal media (yeast nitrogen base without amino acid for *Y. lipolytica*), provided 5 g/L ^13^C_1,2_ glucose. Then, the subcultures were inoculated 1% v/v as free cell culture or hydrogel-immobilized culture in respective minimal media with same labeled glucose. The resulting biomass was harvested in the exponential phase by centrifuge. The biomass protein was washed then hydrolyzed with 6 N HCL at 100 °C for 24 h. A TMDMS (*N*-(tert-butyldimethylsilyl)-*N*-methyltrifluoroacetamide) method was used to analyze the proteinogenic amino acid labeling profiles via GC-MS ^39^.

## Supplementary Information

Supplementary Information.

## Data Availability

The datasets used and/or analyzed during the current study available from the corresponding authors on reasonable request.
