# Supplementary material for: Photobiological production of high-value pigments via compartmentalized co-cultures using Ca-alginate hydrogels
Source: Sci Rep. 2022 Dec 22;12:22163. doi: 10.1038/s41598-022-26437-y (PMC9780300; doi:10.1038/s41598-022-26437-y)
Supplement: Supplementary file 1 — Supplementary Information. [file 41598_2022_26437_MOESM1_ESM.docx]

**Supplementary Material**

**Table S1. Strains used in this study**

| Strain name | Genome type | Reference |
| --- | --- | --- |
| Engineered *Yarrowia lipolytica* | *Y. lipolytica* CLIB138 YALrDNA-URA3-TEF-carB-carRP | ^1^ |
| JBEI-137184a (TEAM-1120) | KT2440 *Pseudomonas putida*_5402::arap-Sc.bpsA,Bc.sfp | ^2^ |
| 2973-cscB | *Synechococcus elongatus* 2973 NS3::P_lacUV5_-cscB | ^3^ |
| SL2234 | *E. coli* top10 cells P_trc10_ promoter expressing eYFP PpMQAK1 vector^4^ | This study |

**Supplementary Figure 1.** Standard curves of β-carotene (A) and indigoidine (B) built in-house.

B.

A.

**Supplementary Figure 2** *P. putida/S. elongatus* co-culture fluorescent image


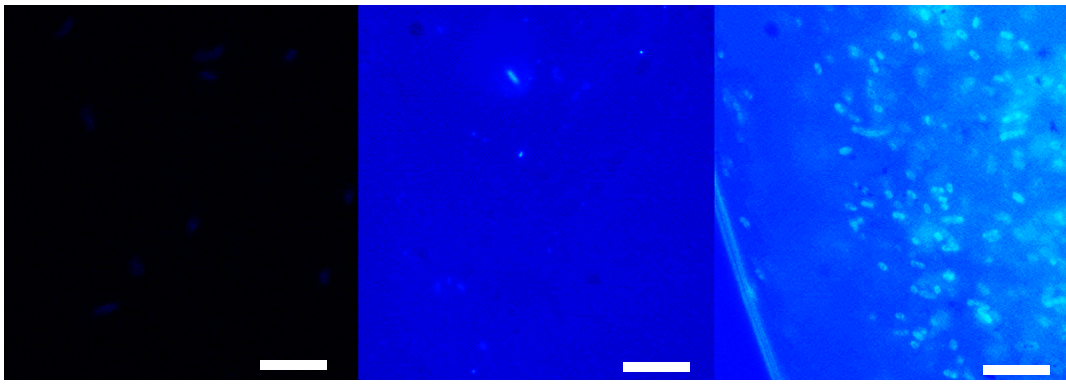

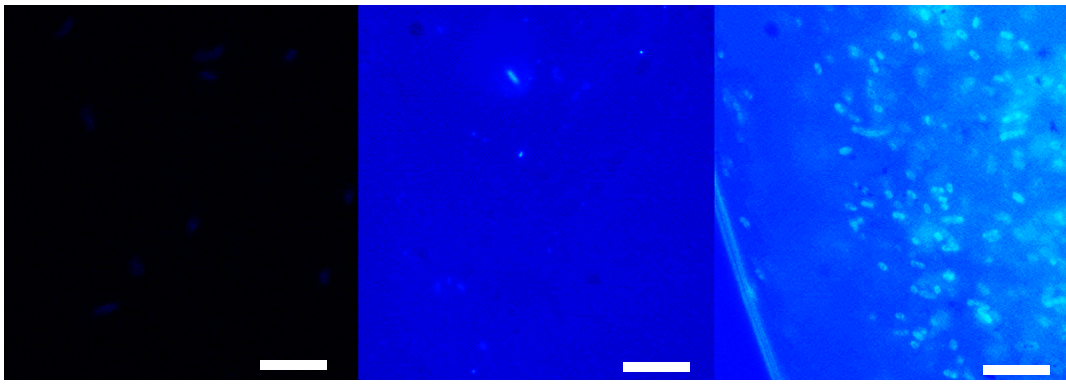


**Hydrogel co-culture**

*P. putida*

Hydrogel

boundary

**Free cell co-culture**

Fluorescent microscope image from 4',6-diamidino-2-phenylindole (DAPI) filter. From left to right images demonstrate free cell co-culture and hydrogel co-culture of *P. putida* and *S. elongatus*, 100 × magnification. Scale bars are 10 µm.

**Table S2. GC-MS 13C labeled amino acid from biomass (m/z = 57)**

| Amino acid | Labeling | labeled proportion | |
| --- | --- | --- | --- |
|  |  | free cell biomass | hydrogel biomass |
| ala | m+0 | 0.55 | 0.54 |
| ala | m+1 | 0.15 | 0.14 |
| ala | m+2 | 0.30 | 0.33 |
| asp | m+0 | 0.37 | 0.36 |
| asp | m+1 | 0.30 | 0.30 |
| asp | m+2 | 0.25 | 0.25 |
| asp | m+3 | 0.09 | 0.09 |
| glu | m+0 | 0.26 | 0.26 |
| glu | m+1 | 0.27 | 0.26 |
| glu | m+2 | 0.29 | 0.29 |
| glu | m+3 | 0.13 | 0.13 |
| glu | m+4 | 0.06 | 0.06 |
| gly | m+0 | 0.66 | 0.67 |
| gly | m+1 | 0.34 | 0.33 |
| leu | m+0 | 0.21 | 0.21 |
| leu | m+1 | 0.23 | 0.23 |
| leu | m+2 | 0.27 | 0.27 |
| leu | m+3 | 0.18 | 0.18 |
| leu | m+4 | 0.08 | 0.08 |
| leu | m+5 | 0.03 | 0.03 |
| met | m+0 | 0.30 | 0.31 |
| met | m+1 | 0.32 | 0.32 |
| met | m+2 | 0.23 | 0.23 |
| met | m+3 | 0.12 | 0.12 |
| met | m+4 | 0.02 | 0.02 |
| phe | m+0 | 0.25 | 0.25 |
| phe | m+1 | 0.15 | 0.15 |
| phe | m+2 | 0.31 | 0.31 |
| phe | m+3 | 0.13 | 0.13 |
| phe | m+4 | 0.11 | 0.11 |
| phe | m+5 | 0.03 | 0.03 |
| phe | m+6 | 0.01 | 0.01 |
| phe | m+7 | 0.00 | 0.00 |
| phe | m+8 | 0.00 | 0.00 |
| ser | m+0 | 0.54 | 0.53 |
| ser | m+1 | 0.22 | 0.22 |
| ser | m+2 | 0.24 | 0.25 |
| tyr | m+0 | 0.25 | 0.25 |
| tyr | m+1 | 0.16 | 0.15 |
| tyr | m+2 | 0.30 | 0.30 |
| tyr | m+3 | 0.13 | 0.13 |
| tyr | m+4 | 0.11 | 0.11 |
| tyr | m+5 | 0.03 | 0.03 |
| tyr | m+6 | 0.01 | 0.01 |
| tyr | m+7 | 0.00 | 0.00 |
| tyr | m+8 | 0.00 | 0.00 |
| val | m+0 | 0.34 | 0.33 |
| val | m+1 | 0.16 | 0.16 |
| val | m+2 | 0.34 | 0.34 |
| val | m+3 | 0.09 | 0.09 |
| val | m+4 | 0.08 | 0.08 |
| lys | m+0 | 0.17 | 0.16 |
| lys | m+1 | 0.27 | 0.26 |
| lys | m+2 | 0.28 | 0.28 |
| lys | m+3 | 0.18 | 0.19 |
| lys | m+4 | 0.08 | 0.08 |
| lys | m+5 | 0.02 | 0.02 |
| his | m+0 | 0.07 | 0.06 |
| his | m+1 | 0.47 | 0.47 |
| his | m+2 | 0.33 | 0.36 |
| his | m+3 | 0.10 | 0.07 |
| his | m+4 | 0.02 | 0.01 |
| his | m+5 | 0.02 | 0.01 |

**Supplementary Material References**

1. Czajka, J.J. et al. Engineering the oleaginous yeast Yarrowia lipolytica to produce the aroma compound beta-ionone. *Microb Cell Fact* **17**, 136 (2018).

2. Banerjee, D. et al. Genome-scale metabolic rewiring improves titers rates and yields of the non-native product indigoidine at scale. *Nature Communications* **11**, 5385 (2020).

3. Lin, P.C., Zhang, F. & Pakrasi, H.B. Enhanced production of sucrose in the fast-growing cyanobacterium Synechococcus elongatus UTEX 2973. *Sci Rep* **10**, 390 (2020).

4. Huang, H.H., Camsund, D., Lindblad, P. & Heidorn, T. Design and characterization of molecular tools for a Synthetic Biology approach towards developing cyanobacterial biotechnology. *Nucleic Acids Res* **38**, 2577-2593 (2010).
